# A New Kind of Acarus

**Published:** 1867-08

**Authors:** 


A New Kind of Acarus.—M. Indée has noticed among the Kabyles of Northern Africa a pruriginous complaint somewhat like the itch; where, however, the acarus, forming a black spot on the skin, moving about with energy, is different from the well-known acarus scabiei. The sulphuro-alkaline ointment destroyed the animalcule. In France, M. Rouyer has noticed in the department of Indre, a popular pruriginous eruption, affecting the country people who had handled the wheat somewhat spoiled by the frequent rains of last summer. The same parisites were here observed.—*Lancet.*

